# Super-Macroporous Pulluan Cryogels as Controlled Active Delivery Systems with Controlled Degradability

**DOI:** 10.3390/mi14071323

**Published:** 2023-06-28

**Authors:** Betul Ari, Mehtap Sahiner, Selin Sagbas Suner, Sahin Demirci, Nurettin Sahiner

**Affiliations:** 1 Department of Chemistry, Faculty of Science, Canakkale Onsekiz Mart University, Terzioglu Campus, 17100 Canakkale, Turkey; betullcan@gmail.com (B.A.); sagbasselin@gmail.com (S.S.S.); sahindemirci@gmail.com (S.D.); 2 Bioengineering Department, Faculty of Engineering, Canakkale Onsekiz Mart University, Terzioglu Campus, 17100 Canakkale, Turkey; msahiner@comu.edu.tr; 3 Nanoscience and Technology Research and Application Center (NANORAC), Canakkale Onsekiz Mart University, Terzioglu Campus, 17100 Canakkale, Turkey; 4 Department of Chemical and Biomolecular Engineering, Faculty of Engineering, University of South Florida, Tampa, FL 33620, USA; 5 Department of Ophthalmology, Morsani College of Medicine, University of South Florida, 12901 Bruce B. Downs Blvd, MDC21, Tampa, FL 33612, USA

**Keywords:** biocompatible/degradable polysaccharide cryogel, pullulan cryogel, natural polymeric cryogel drug carrier, ciprofloxacin release

## Abstract

Here, super-macroporous cryogel from a natural polysaccharide, pullulan was synthesized using a cryo-crosslinking technique with divinyl sulfone (DVS) as a crosslinker. The hydrolytic degradation of the pullulan cryogel in various simulated body fluids (pH 1.0, 7.4, and 9.0 buffer solutions) was evaluated. It was observed that the pullulan cryogel degradation was much faster in the pH 9 buffer solution than the pH 1.0 and 7.4 buffer solutions in the same time period. The weight loss of the pullulan cryogel at pH 9.0 within 28 days was determined as 31% ± 2%. To demonstrate the controllable drug delivery potential of pullulan cryogels via degradation, an antibiotic, ciprofloxacin, was loaded into pullulan cryogels (pullulan-cipro), and the loading amount of drug was calculated as 105.40 ± 2.6 µg/mg. The release of ciprofloxacin from the pullulan-cipro cryogel was investigated in vitro at 37.5 °C in physiological conditions (pH 7.4). The amount of drug released within 24 h was determined as 39.26 ± 3.78 µg/mg, which is equal to 41.38% ± 3.58% of the loaded drug. Only 0.1 mg of pullulan-cipro cryogel was found to inhibit half of the growing *Escherichia coli* (*E. coli*) and *Staphylococcus aureus* (*S. aureus*) colonies for 10 min and totally eradicated within 2 h by the release of the loaded antibiotic. No significant toxicity was determined on L929 fibroblast cells for 0.1 mg drug-loaded pullulan cryogel. In contrast, even 1 mg of drug-loaded pullulan cryogel revealed slight toxicity (e.g., 66% ± 9% cell viability) because of the high concentration of released drug.

## 1. Introduction

Cryogels have superior properties with respect to common hydrogels in terms of fast response to changes in the environment conditions including pH, temperature, and ionic strength, in addition to elasticity and great mechanical strengthm due to their interconnected super-macroporous structures [1,2,3]. In the literature, many cryogels have been reported from synthetic monomers and polymers such as hydroxyethyl methacrylate (HEMA), acrylamide (AAm), and polyvinyl alcohol (PVA), along with some from natural polymers [4,5]. The cryogels prepared from natural structures afford many advantages due to their sustainable, degradable, and biocompatible structures; thus, they have attracted great attraction in recent years [4,5].

Natural polymers that can be obtained from renewable and easily accessible sources are intriguing materials to be employed in cryogel preparation. In particular, polysaccharide-based cryogels have become versatile materials in biomedical applications due to their biocompatible, nontoxic, and even biodegradable structures [6]. Polysaccharides can be categorized according to their chemical structure as acidic (i.e., alginic acid, hyaluronic acid, chondroitin sulfate, and carrageenan), basic (e.g., chitin and chitosan), or neutral (i.e., agarose, dextran, and pullulan) structures [7,8,9,10]. Pullulan, a neutral polysaccharide, consists of maltotriose residues linked by α-1,4 and α-1,6 glycosidic bonds, attained via the microorganism *Aureobasidium pullulans*, especially found in industrial wastewater. Pullulan polysaccharide is an important biomolecule in the preparation of polysaccharide-derived hydrogels, due to its hydrophilic, biodegradable, and biocompatible nature, as well as the availability of functional groups (hydroxyl) suitable for further modification [11,12,13,14]. Pullulan has several benefits as a drug delivery system; it is non-toxic, has low immunogenicity, and exhibits high-water solubility [15,16]. Many studies have investigated pullulan. For example, Iswariya et al. prepared collagen–pullulan hydrogels crosslinked with sodium trimetaphosphate for skin reconstruction [17]. Through in vitro and in vivo studies, they revealed that porous collagen–pullulan hydrogels are promising biomaterials for wound-healing applications [17]. In another study, Han et al. used chemically crosslinked pullulan/gelatin-based nanogels as extracellular matrix-like scaffolds in tissue engineering [18]. In addition, it was reported that pullulan-based nanogels are nanomaterials with a high potential for use as active agent carrier agents [18]. In another study with pullulan, Bae et al. synthesized methacrylate pullulan (PulMA) hydrogels and demonstrated their use in cell culture and tissue engineering applications [19]. They reported that PulMA hydrogels can be used for applications where controlled cell proliferation is important, especially hepatic and embryonic stem-cell cultures. In addition, they prepared PulMA/GelMA hydrogels with improved biological properties by combining PulMA hydrogels with methacrylate gelatin (Gel/MA) [19]. In addition to these studies, it has been reported that the use of pullulan-based materials is not limited to biomedical and pharmaceutical applications; they are also used in other applications such as food, cosmetics, and the environment [20,21,22,23,24]. It has been reported that films prepared from pullulan are colorless, transparent, and edible; thus, they can be used as food packaging materials [23,24]. Moreover, it has been stated that pullulan can be used in cosmetic products such as shampoo, cream, and makeup due to its high water-holding capacity, nontoxicity, and nonirritant nature to the human body [20,21]. In addition, Hezarkhani et al. prepared pullulan/poly(N-vinylimidazole) (PNVI) cryogels for use in environmental applications, reporting that these cryogels can be used as effective dye adsorbents for wastewater treatment [22]. Furthermore, hybrid cryogels made of pullulan and poly(N-vinyl imidazole) were created using free-radical polymerization and chemical crosslinking in an alkaline solution containing ammonium persulfate, epichlorohydrin, pullulan, and NVI [25]. These composite cryogels were utilized to absorb dyes in wastewater treatment. A scaffold made of dual-crosslinked pullulan/gelatin cryogel was created through a process of crosslinking oxidized pullulan-gelatin using the Schiff’s base reaction, followed by cryogelation [26]. Pullulan/dopamine cryogels were synthesized for wound dressings with hemostatic properties by providing periodate-oxidized pullulan [27]. Cryogels made from pullulan and polyHEMA were created using crosslinkers such as epichlorohydrin and ethylene glycol dimethacrylate for cell binding and proliferation [28]. Although pullulan-based composite cryogels have been prepared in the literature, there are no reports of cryogel based solely on pullulan polysaccharide.

In this study, super-porous pullulan cryogels were prepared and optimized using the cryo-polymerization technique with different molar ratios of divinyl sulfone (DVS) crosslinker based on repeating units of pullulan. The swelling properties of the prepared pullulan cryogels in DI water, such as maximum percentage swelling, percentage moisture content, percentage porosity, and pore volume, were investigated. In order to explore the potential use of pullulan cryogels in the biomedical field, their hydrolytic degradation behavior in different pH environments corresponding to body fluids were investigated. In addition, degradable pullulan cryogel was loaded with an antibiotic (ciprofloxacin), and the drug release profile of the drug-loaded cryogel was investigated in vitro at 37.5 °C (approximate body temperature) and pH 7.4 (physiological conditions). In addition, the cytotoxicity of the prepared pullulan cryogels was investigated using the MTT test to highlight the biosafety of these materials in biomedical applications as a drug carrier material.

## 2. Materials and Methods

### 2.1. Materials

Pullulan (Alfa Aesar, Great Britain, Heysham, UK) and divinyl sulfone (DVS, 98%, Merck, Japan) were used in the synthesis of pullulan cryogels. Ciprofloxacin (Recordati, Pharmaceuticals, Istanbul, Turkey) was used as a model drug in the drug release studies. Gram-negative bacterium *Escherichia coli* (ATCC 8739) and Gram-positive bacterium *Staphylococcus aureus* (ATCC 6538) were obtained from KWIK-STIK, Microbioloics. Nutrient agar (NA, Condolab, Madrid, Spain) as a solid growth medium and nutrient broth (NB, Merck, Darmstadt, Germany) as a liquid medium were used as received. As the cell culture, the L929 fibroblast cell line (Mouse C3/An connective tissue) was obtained from the SAP Institute, Ankara, Turkey. Dulbecco’s modified Eagle’s medium (DMEM) (4500 mg/L glucose, 3.7 g/L sodium pyruvate, and 0.5 g/mL L-glutamine) as the cell culture medium, fetal bovine serum (FBS, heat inactivated), and trypsin-EDTA (0.25%) were purchased from PanBiontech (A GmbH, Aidenbach, Germany). For the calorimetric cell viability analysis, 3-(4,5-dimethylthiazol-2-yl)-2,5-diphenyltetrazolium bromide (MTT) was obtained from neoFroxx (A GmbH, Hesse, Germany) and trypan blue (0.5% solution) was obtained from Biological Industries. Dimethyl sulfoxide (DMSO, 99.9%, Carlo-Erba, Val-de-Reuil, France) was used in the cytotoxicity studies.

### 2.2. Synthesis of Super-Macroporous Pullulan Cryogels

In the synthesis of pullulan cryogels, the method that we used in the synthesis of different biopolymer cryogels was employed by changing a few parameters [29]. Accordingly, 0.1 g of pullulan biopolymer was added to 5 mL of 0.5 M NaOH aqueous solution and stirred at 500 rpm for 1 h until dissolved. Then, the prepared pullulan solution was cooled in a deep freezer at −20 °C for 3 min. The chilled pullulan solution was mixed with 200 mol.% DVS with respect to the repeated unit of the pullulan biopolymer (C_22_H_38_O_15_, 542 g/mol), mixed at 100 rpm for 30 s, placed in plastic pipettes with a diameter of ~0.6 mm and placed in a deep freezer at −20 °C for cryo-crosslinking. The reaction proceeded for 24 h. Finally, the prepared pullulan cryogels were cut to similar sizes (~0.5 cm) and washed with distilled water to remove unreacted reagents. During the washing process with pure water, the washing water was changed every 2 h, and the washing process was continued for 8 h. The washed cryogels were dried using a freeze-dryer. These cryogels were stored in lidded containers for further processing.

### 2.3. Characterization of Super-Macroporous Pullulan Cryogels

Morphological structures of pullulan cryogel were determined using scanning electron microscopy (SEM, QUANTA 400F Field Emission). To obtain SEM images of pullulan cryogels, the sample was coated with Au/Pd before analysis, and SEM images were obtained under 20 kV voltage.

Swelling behaviors of cryogels such as the maximum swelling ratio (%S), moisture content (%M), and porosity (%P) were investigated [26]. A piece of dried cryogel of known weight was transferred to a container with distilled water and allowed to swell at room temperature. Swollen cryogels were removed from the distilled water, surface water was blot-dried using filter paper, and the cryogel pieces were weighed again. The %S and %M values of cryogels were calculated using Equations (1) and (2). In order to establish the porosity of the cryogels, the same swollen cryogels were reweighed while pinching with two fingers, and the %P values were calculated using Equation (3).

%S = [(m_s_ − m_d_)/m_d_] × 100, (1)

%M = [(m_s_ − m_d_)/m_s_] × 100, (2)

%P = [(m_s_ − m_sq_)/m_s_] × 100, (3)

where m_s_, m_d_, and m_sq_ represent the weights of the swollen, dry, and compressed cryogel pieces, respectively.

To determine the pore volume (V_p_) of the cryogels, a dry piece of cryogel of known weight was weighed after swelling in cyclohexane. Then, the pore volume of the cryogels was calculated using Equation (4).

V_p_ = (m_ch_ − m_d_)/(m_d_ × d_ch_), (4)

where m_ch_ represents the weight of cryogel blown in cyclohexane, m_d_ represents the weight of dry cryogel, and d_ch_ represents the density of cyclohexane [26].

The pullulan biopolymer and pullulan cryogel were structurally characterized by Fourier-transform infrared (FT-IR, Thermo, Waltham, MA, USA) spectroscopy analysis. The analyses were recorded using the ATR technique in a wavelength range of 4000–650 cm^−1^ with a discrimination power of 4 cm^−1^.

The thermal degradation behaviors of pullulan biopolymer and pullulan cryogel were determined with the help of thermograms obtained as a result of heating the samples up to 750 °C in the presence of 20 mL/min N_2_ gas flow using thermogravimetric analysis (TGA, SII, Seiko, Japan).

### 2.4. The Hydrolytic Degradation of Super-Macroporous Pullulan Cryogels

The hydrolytic degradation behavior of pullulan cryogel was investigated gravimetrically. Briefly, cryogel weighing approximately 15 mg was placed in tubes containing 15 mL of different pH buffer solutions (stomach pH value of ~1.0, physiological pH value of ~7.4, and duodenum pH value of ~9.0) and allowed to swell for 10 min. Then, the weight of the swollen cryogel was recorded and again put back into the tubes. The tubes were placed in a shaking water bath at 37.5 °C. The percentage weight loss of cryogels over time was calculated using Equation (5) by weighing the swollen pullulan cryogels at certain times [29]. Hydrolytic degradation studies were repeated three times to evaluate the accuracy, and the mean values were reported by calculating the standard deviations.

%Weight loss = [100 − (m_t_/m_i_) × 100], (5)

where m_t_ represents the weight of the cryogel at time t, and m_i_ represents the initial weight of the swollen cryogel.

### 2.5. Drug Loading and Drug Release Study of Super-Macroporous Pullulan Cryogels

Ciprofloxacin as an antibiotic drug was loaded into the pullulan cryogel via absorption. Firstly, 50 mL of a 5000 ppm ciprofloxacin aqueous drug solution was prepared. To load the drug into the pullulan cryogel, an approximately 0.2 g piece of cryogel was put into the drug solution and left for 24 h. At the end of the drug loading period, the drug-loaded cryogels were freeze-dried without any washing and stored at +4 °C in enclosed tubes until use. The drug loading amount (µg/mg) was calculated using Equation (6).

Drug loading amount = [(C_0_ − C_t_) × V]/m_c_, (6)

where “C_0_” (µg/L) is the initial concentration of the drug solution, “C_t_” (µg/L) is the concentration of the drug solution at the end of the 24 h reaction, “V” (L) is the volume of the drug solution, and “m_c_” (mg) is the weight of the pullulan cryogel.

For drug release studies, drug-loaded cryogel pieces weighing about 30 mg were transferred into flasks containing 250 mL of pH 7.4 PBS. The drug release profile of drug-loaded pullulan cryogel was determined using a UV/Vis spectrophotometer (SP-UV300SRB, Spectrum, Quanzhou, China) according to a calibration curve previously prepared in a pH 7.4 PBS solution with ciprofloxacin. The calibration curve was constructed for ciprofloxacin at a wavelength of 266 nm. Drug release studies were performed in triplicate to ensure accuracy. The results are presented as mean values with standard deviations. The drug release amount (µg/mg) and drug release capacity (%) were calculated using Equations (7) and (8), respectively.

Drug release amount = (C_t_ × V)/m_c_, (7)

where C_t_ (µg/L) is the concentration at time t of the PBS solution containing the drug-loaded (pullulan-cipro) cryogel, V (L) is the volume of pH 7.4 PBS, and m_c_ (mg) is the weight of the drug-loaded (pullulan-cipro) cryogel.

Drug release capacity % = (drug release amount/drug loading amount) × 100.(8)

### 2.6. Antimicrobial Activity of Drug Loaded Pullulan-Cipro Cryogels

The antimicrobial activity of drug-loaded pullulan-cipro cryogels was evaluated using a broth macro-dilution test against Gram-negative bacterium *E. coli* and Gram-positive bacterium *S. aureus*, as described in the literature [30]. Stock bacterial suspensions of *E. coli* and *S. aureus* strains were adjusted to a McFarland standard of 0.5 (1 × 10^8^ CFU/mL). Then, 100 μL of bacterial suspension (0.5 McFarland) was added to 10 mL of nutrient broth (NB) as a liquid growth medium in tubes. The pullulan-cipro cryogel was sterilized under UV irradiation for 5 min prior to the test. Next, 0.1 mg of pullulan-cipro cryogel was placed in 10 mL of bacterium-containing growth medium. As a negative control, 100 μL of bacterial suspension (0.5 McFarland) was suspended in 10 mL of NB without drug-loaded cryogel. At different incubation times (0, 0.16, 0.5, 1, 1.5, 2, 4, and 6 h), 100 μL of the bacterial suspension was taken from these tubes and seeded onto nutrient agar solid growth medium after dilution with 0.9 NaCl solution at 100-fold. Then, the plate was incubated at 35 °C for 24 h; the living bacterial colonies were counted, and the number was compared with the control group to determine the bacterial cell viability %. The test was carried out in triplicate, and the results are reported with standard deviations.

### 2.7. Cytotoxicity of Pullulan and Drug-Loaded Super-Macroporous Pullulan Cryogels

The cytotoxicity of the pullulan polysaccharide, ciprofloxacin antibiotic, prepared pullulan cryogel, and drug-loaded pullulan-cipro cryogel was analyzed on the L929 human fibroblast cell line. Before the analysis, the samples were sterilized under UV irradiation at 420 nm for 5 min. Separately, the cells were grown in a CO_2_/air atmosphere (5% CO_2_/95% air atmosphere) with DMEM growth medium supplemented with 10% FBS and 1% antibiotic at 37 °C for 7 days to obtain 80% cell confluency. Then, fibroblasts were counted; approximately 5 × 10^4^ cells/well were seeded into a 96-well plate and incubated in a CO_2_/air atmosphere at 37 °C for 24 h. Next, the old medium was removed, and 100 μL of 0.05–1 mg/mL pullulan and ciprofloxacin solution dissolved in growth medium was added to the cells as a positive control. Only 100 μL of growth medium was added to the attached cells in the wells as a negative control. Separately, pullulan cryogel and drug-loaded pullulan-cipro cryogel weighing 0.1–1 mg were carefully placed on the cells in the wells, along with 100 μL of growth medium. The plate was incubated for 24 h in the same conditions as used for cell growthg. At the end of the incubation, the samples and media in the well plate were discarded, and the wells were washed with sterile phosphate-buffered saline (PBS). The in vitro cell viability of fibroblasts in the presence of the samples were determined using the MTT colorimetric method according to the measurement of the intracellular formazan production of living cells [31]. For this, 100 µL of 0.5 mg/mL MTT solution (prepared in 1 mL of PBS at 5 mg/mL and diluted with 9 mL of DMEM) was added to the wells, and the 96-well plate was incubated at 37 °C for 3 h in dark. Following this period, the MTT solution was removed, and 200 µL of DMSO was added to the wells to dissolve the produced formazan crystals. Lastly, the well plate was shaken to homogenize the color, and the optical density was measured using a plate reader (Multiskan™ FC, Microplate Photometer) at 570 nm. The test was repeated in triplicate, and the results are reported with standard deviations.

### 2.8. Statistical Evaluation

Using GraphPad Prism software, the statistical analysis of the cumulative weight loss and antimicrobial activity results of pullulan cryogels was performed using Dunnett’s multiple-comparison test and one-way ANOVA. For the hydrolytic degradation test, the statistical differences of pullulan cryogel at different pH conditions were compared with the degradation result at pH 7.4 for 28 days of incubation time. Similarly, the bacterial viability results were compared with the negative control group, i.e., a bacterial suspension without drug-loaded cryogel. In the cytotoxicity analysis, the obtained cell viability results were compared with the control group, and statistical differences were determined using Student’s *t*-test with GraphPad Prism software. A *p*-value <0.05 was considered statistically significant.

## 3. Results

### 3.1. Pullulan Cryogels as Super-Macroporous Structures

**Figure 1:**
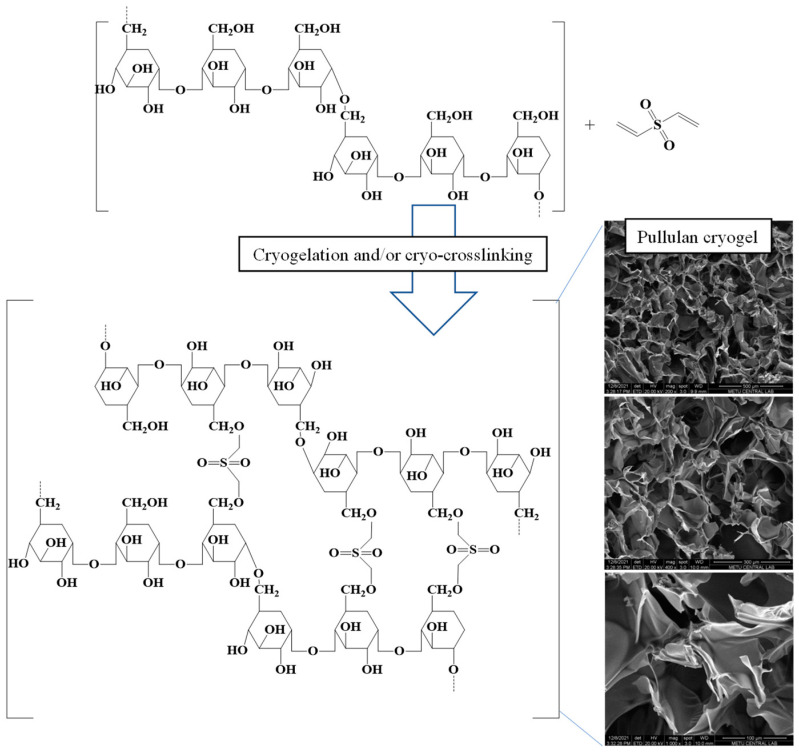
Schematic representation of synthesis (**left**); SEM images of pullulan cryogels (**right**).

The synthesis of pullulan cryogels was carried out at temperatures below the freezing point of the solvent (water) used, i.e., cryogenic conditions, involving cryo-crosslinking reactions of the precursor molecule (pullulan) and the crosslinker (DVS) in the condensed unfrozen liquid phase. The reaction took place within the ice crystals; after the ice crystals melted in room conditions, interconnected super-porous cryogels were obtained. The chemical structures of pullulan and DVS crosslinker, along with the SEM images of prepared pullulan cryogels, are given in Figure 1.

The SEM images of pullulan cryogels were taken at different magnifications. As shown in Figure 1, the pullulan cryogels had interconnected pores ranging from 1 to 200 µm. The material porosity is extremely important in drug transport and release applications. The porosity of the prepared pullulan cryogels could be readily controlled as a function of the amounts of precursors (pullulan polymer, crosslinker, water, etc.) [29,32].

The swelling (S%), porosity (P%), and moisture content (M%) percentages and the pore volume values (V_P_) for the prepared pullulan cryogel are summarized in Table 1.

Accordingly, the percentage swelling of the pullulan cryogel was found to be 588% ± 22%. However, for pullulan cryogel, the percentage porosity value was calculated as 75% ± 0.3%, the percentage moisture content was calculated as 85% ± 0.5%, and the pore volume was calculated as 5.0 ± 0.2 mL/g.

**Figure 2:**
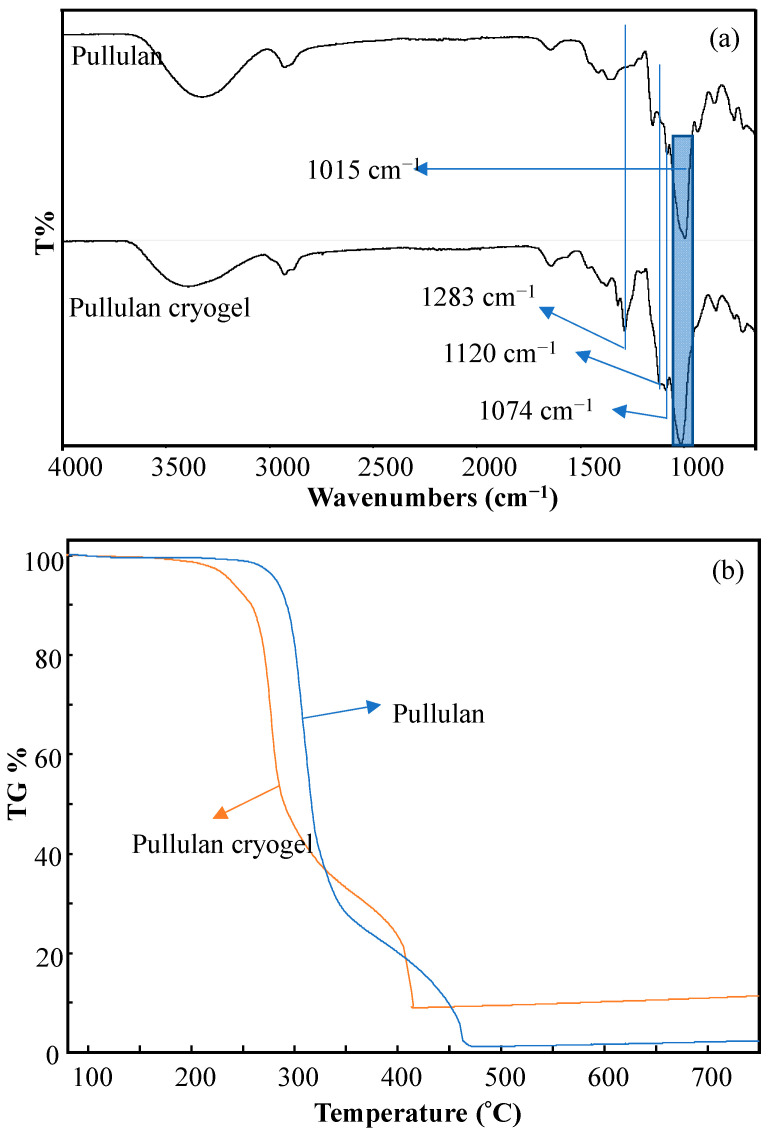
(**a**) FT-IR spectra and (**b**) thermal degradation curves of pullulan biopolymer and pullulan cryogel.

The FT-IR spectra and thermal stability of pullulan biopolymer and pullulan cryogel are compared in Figure 2a,b, respectively. According to the comparison of the FT-IR spectra of the pullulan biopolymer and the pullulan cryogel in Figure 2a, the –OH peaks observed at 1015 cm^−1^ were attributed to the hydroxyl groups in the pullulan molecule, appearing sharply in both spectra. In addition, the peaks from the DVS crosslinker, which were attributed to symmetrical and asymmetrical O–S–O and S=O peaks, were observed at 1283, 1120, and 1074 cm^−1^ in the FT-IR spectrum of pullulan cryogels due to the crosslinking with DVS. These observed peaks showed that pullulan cryogels were synthesized successfully.

According to the TGA thermograms in Figure 2b, the pullulan biopolymer lost approximately 75% of its mass between 300 and 350 °C, and approximately 98% of its total mass in the 360–560 °C range, thermally decomposing at the second step. The significant weight loss of 75%, specific to an exothermic phenomenon, observed in the 300–360 °C range was consistent with the literature [29,33,34]. This phenomenon is closely in line with earlier research and can be explained by the depolymerization and decomposition of the polymeric matrix at higher temperatures [35,36]. The pullulan cryogel, on the other hand, started to decompose at 200 °C and lost about 60% of its mass up to 300 °C, before losing approximately 90% of its total mass in a second thermal decomposition in the temperature range 350–420 °C. It was observed that the thermal stability of pullulan cryogels was relatively low compared to the pullulan molecule, and this result was also compatible with the reported thermal behavior of other polysaccharide-based cryogels such as dextran and p(β-cyclodextrin) cryogels [29,33].

### 3.2. The Degradability of Super-Macroporous Pullulan Cryogels

It is crucial that biomaterials are degradable in the presence of body fluids or enzymes, and they should be eliminated from the body after executing their functions without any accumulation [37]. Thus, hydrolytically or enzymatically degradable biomaterials with controlled degradability play an important role in drug release. Various factors such as swelling properties, porosity, type and chemical content of crosslinker, and pH and temperature of the medium can affect the hydrolytic degradation ability of the materials [38,39,40]. The hydrolytic degradation behavior of crosslinked structures can be easily controlled by changing various parameters such as porosity, as well as the type and amount of crosslinker used [29,41]. The hydrolytic degradation behavior of pullulan cryogels, which are intended to be used as drug delivery systems in the biomedical field, were investigated in various buffer solutions at pH 1.0 (approximate stomach pH), pH 7.4 (physiological pH), and pH 9.0 (approximate duodenal pH).

**Figure 3:**
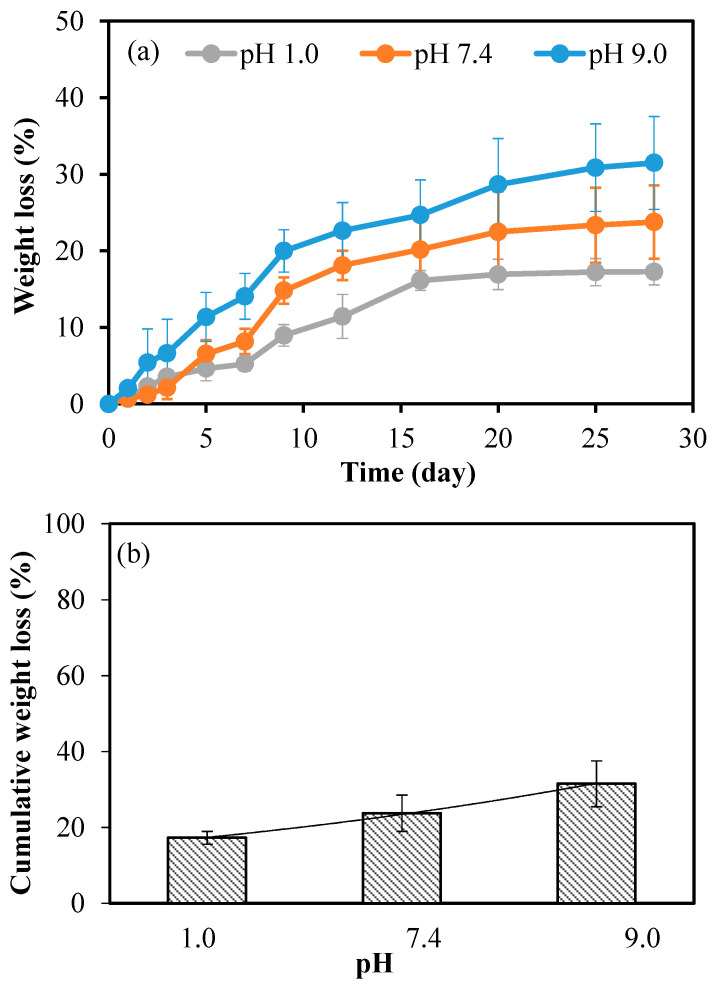
Hydrolytic degradation behavior of pullulan cryogel in various buffer solutions: (**a**) weight loss (%) versus time; (**b**) cumulative weight loss (%) at 37.5 °C. The statistical analysis of cumulative weight loss (%) in different pH conditions was compared with the cumulative weight loss (%) at pH 7.4, and no significant statistical difference was detected.

The hydrolytic degradation was performed gravimetrically over time as a function of the weight loss of pullulan cryogel, and the results are given in Figure 3a. According to the results, it can be observed that the degradation behaviors of the pullulan cryogel at all solution pH followed a similar trend. The pullulan cryogel degraded almost linearly over 16 days with weight losses of 16% ± 1% and 20% ± 5% at pH 1.0 and pH 7.4, respectively. This was followed by a slow degradation profile up to 28 days for pH 1.0 and pH 7.4, with total weight losses of 17% ± 2% and 24% ± 5%, respectively. On the other hand, pullulan cryogel exhibited a somewhat faster and more degradable profile at pH 9.0. At pH 9.0, degradation was almost linear up to 20 days with a weight loss of 29% ± 6%. After 28 days, the weight loss reached 31% ± 2% and then persisted with a very slight degradation profile.

Figure 3b shows the cumulative weight loss of hydrolytic degradation of the pullulan cryogel in different pH conditions at the end of the 28th day. These weight losses, for pH 1.0, 7.4, and 9.0, were 17% ± 2%, 24% ± 5%, and 31% ± 6%, respectively. According to the statistical analysis of these degradation results at different pH with pH 7.4, no significant difference was found. The hydrolytic degradation results show that the pullulan cryogel possessed a higher degradation profile under basic conditions compared to acidic or neutral conditions. The pullulan cryogel degraded more under these conditions because it had higher swelling rates in basic conditions. This result clearly shows that the swelling rate and degradation behavior of pullulan cryogel could be controlled by the pH value.

In the literature, the hydrolytic degradation of pullulan/ECH, pullulan/ECH/polyHEMA, and pullulan/ECH/polyHEMA/polyEGDMA cryogels at pH 2, 7.4, and 12 was investigated by Ustürk et al. [28]. All cryogels were reported to show the highest percentage weight loss in neutral conditions (pH 7.4). They reported that, among these three cryogels, the pullulan/ECH cryogel exhibited a weight loss percentage of over 40% in 3 days at pH 7.4, and this value was calculated as 14% for the pullulan/ECH/polyHEMA/polyEGDMA cryogel. In another study, it was reported that platelet lysate (PL)/oxidized dextran (OD) (PL/OD) cryogels containing different ratios of OD lost ~10–15% of the dry weight of the cryogels after a 6 month hydrolytic degradation period at pH 7.4 [42]. As can be deduced from the studies in the literature, the rate of hydrolytic degradation changes depending on the nature of the copolymer used, pH of the medium, crosslinker type, and ratio. In accordance with the intended use, the hydrolytic degradation can be readily controlled by the medium pH, by the type of polymer precursor, and by the type of crosslinker and its ratio.

### 3.3. The Use of Super-Macroporous Pullulan Cryogel as Drug Carrier Material

**Figure 4:**
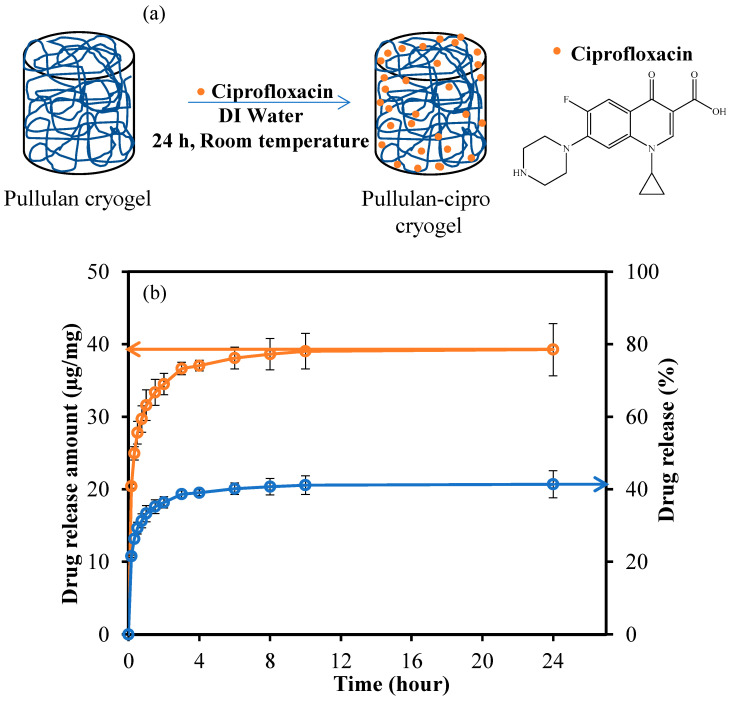
(**a**) Schematic representation of ciprofloxacin drug loading into pullulan cryogel; (**b**) drug release profile of pullulan-cipro cryogel. Drug release conditions: pH 7.4 buffer solution, 37.5 °C.

Drug carrier systems are being continuously engineered to carry drugs throughout the body, enhancing their safety and efficiency [43,44,45,46]. Among these designed systems, there are micro/meso/macroporous and super-porous structures, as well as various sizes of hydrogel such as nanogel [47,48,49], microgel [50,51,52,53], and cryogel [54,55,56,57]. In addition, polymeric structures prepared from synthetic and natural resources are also included in these systems. Biomaterials prepared from natural structures and polymers obtained from renewable and sustainable sources have high potential for use as carrier agents in drug delivery and controlled release systems, due to their natural characteristics such as biocompatibility and biodegradability. Here, the potential of drug-loaded pullulan cryogel for controlled drug release applications was investigated by loading the degradable pullulan cryogel with ciprofloxacin as a model drug via absorption. The physical loading of ciprofloxacin into pullulan cryogel via absorption is presented schematically in Figure 4a.

As the drug was loaded into the pullulan cryogel via adsorption, it was expected that ciprofloxacin would be absorbed relatively more on the outer surface of the cryogel. The frug loading amount (µg/mg), drug release amount (µg/mg), and drug release capacity (%) of pullulan-cipro cryogel were calculated to be 105.40 ± 2.6 (µg/mg), 41.38 ± 3.78 (µg/mg), and 39.26 ± 3.59 (%), respectively. The drug release behavior of ciprofloxacin from the super-porous pullulan-cipro cryogel network as a function of time is presented in Figure 4b as the drug release amount (µg/mg) and drug release (%). The amount of ciprofloxacin released from the super-porous pullulan-cipro cryogel network was calculated as 27.79 ± 1.56 µg/mg after 30 min, and 29.29% ± 1.65% of the drug was released within 30 min in vitro in the physiological medium (pH 7.4). The amount of drug released after 24 h was calculated as 39.26 ± 3.78 µg/mg, which corresponds to about 41.38% ± 3.58% of the total amount of loaded drug. As can be seen, the release of ciprofloxacin from the super-porous pullulan-cipro cryogel network was linear for almost 30 min, and the rate of release dramatically slowed thereafter until 24 h. This result can be explained by the loading of the drug into the cryogel via absorption, leading to a rapid initial release of the drug accumulating on the surface of the cryogel. As a result, it is favorable to design biodegradable pullulan cryogels as drug delivery vehicles in active agent carrier systems. Moreover, the loading of drugs, especially water-soluble drugs, can be accomplished at the desired amount (depending on solubility of the drug) during the synthesis of pullulan cryogels to achieve the desired release amount and release time.

### 3.4. Antibacterial Effect of Drug-Loaded Pullulan-Cipro Cryogels

**Figure 5:**
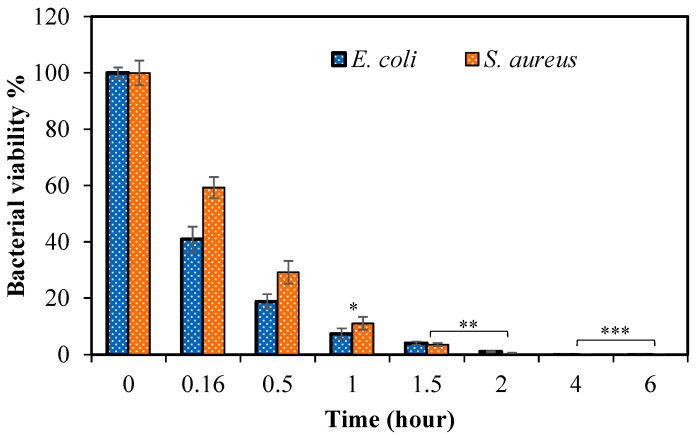
Bacterial viability (%) of Gram-negative *E. coli* and Gram-positive *S. aureus* in the presence of 0.1 mg of drug-loaded pullulan-cipro cryogel over time. * *p* < 0.05, ** *p* < 0.01, and *** *p* < 0.001 compared with control group.

The antimicrobial activity of drug-loaded pullulan-cipro cryogel was assessed against Gram-negative *E. coli* and Gram-positive *S. aureus* strains, which are main causes of nosocomial infections and bacteremia [58]. In addition, these two common strains have various defense mechanisms that render them resistant to many antimicrobials. Therefore, the antibacterial effect of antibiotic-loaded pullulan-cipro cryogel was investigated using the broth macro dilution test against *E. coli* and *S. aureus* bacteria strains, and the results are given in Figure 5.

In the broth macro dilution test, the samples were taken from 10 min to 6 h of ciprofloxacin release from 0.1 mg drug-loaded pullulan-cipro cryogel into 10 mL of NB. As seen in Figure 4, 21.5 ± 0.3 μg/mg cipro was released from pullulan-cipro cryogel within 10 min, and the release gradually continued up to 24 h with 41.3 ± 3.7 μg/mg drug delivered. According to the antibacterial test results, 0.1 mg of the pullulan-cipro cryogels released 2.15 μg of cipro in 10 mL of NB medium, and almost 50% of the bacterial strains were killed within 10 min, as shown in Figure 5. Moreover, 3.6 ± 0.1 μg of the loaded cipro was released from 0.1 mg of pullulan-cipro cryogel in 10 mL of NB for 2 h, and this 0.36 μg/mL cipro release totally inhibited the colony growth of both bacteria within 2 h of incubation time. The minimum bactericidal concentration (MBC) values of the cipro antibiotic were reported as 0.008 and 0.5 μg/mL against *E. coli* and *S. aureus*, respectively, over 24 h of incubation time [59]. Overall, pullulan-cipro cryogel weighing 0.1 mg was significantly effective within just 10 min (0.16 h). Similarly, statistical analysis showed that the bacterial viability was significantly different after 1 h of incubation time. Furthermore, within 2 h of the antibiotic release from the cryogels, both bacterial strains were completely eradicated. Hence, pullulan cryogels with a high capacity for drug loading and release are quite promising, and pullulan-cipro cryogels could be used to decrease the prevalence of healthcare-associated infections, as well as reduce antimicrobial resistance by reducing both the dose and frequency of antibiotic administration.

### 3.5. Cytotoxicity Results of Pullulan and Drug-Loaded Pullulan Cryogels

**Figure 6:**
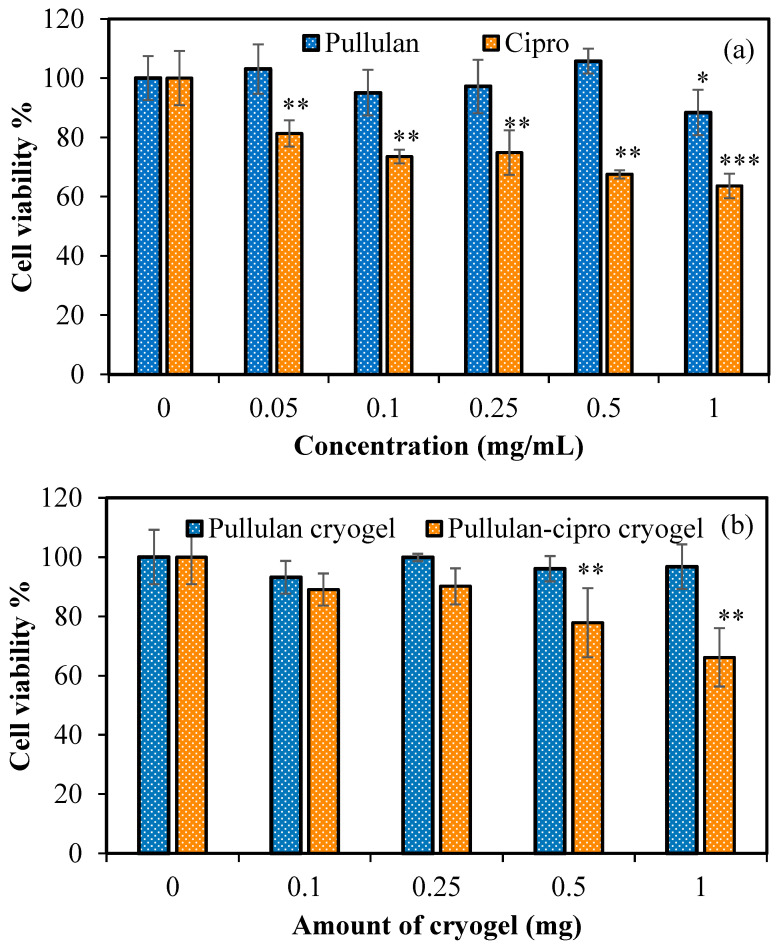
Cell viability of L929 fibroblast cells in the presence of (**a**) pullulan polysaccharide and ciprofloxacin antibiotic at concentrations ranging from 0.05 to 1 mg/mL and (**b**) bare pullulan cryogel and drug loaded pullulan-cipro cryogel at concentrations ranging from 0.1 to 1 mg over 24 h of incubation time. * *p* < 0.05, ** *p* < 0.01, and *** *p* < 0.001 compared with control group.

The cell viability (%) of L929 fibroblasts in contact with pullulan and ciprofloxacin over 24 h of incubation time is given in Figure 6a.

As shown in Figure 6a, pullulan polysaccharide was nontoxic up to 1 mg/mL concentration, but the cipro antibiotic showed less toxicity, even at 0.5 mg/mL concentration, with 81% ± 4% cell viability. Furthermore, the cytotoxicity of cipro was slightly increased upon increasing the drug concentration, and 64% ± 4% cell viability was found for a 1 mg/mL concentration of cipro solution. There was no statistical difference among all concentrations of pullulan polysaccharide and the control (0.05–0.5 mg/mL). Only 1 mg/mL pullulan polysaccharide was statistically different from the control. In the presence of ciprofloxacin drug, cell viability was statistically different from the control at all concentrations (0.05–1 mg/mL). Furthermore, the cytotoxicity of the prepared pullulan cryogel and its drug-loaded form in the 0.1–1 mg cryogel range was tested over 24 h of incubation time, and the results are illustrated in Figure 6b. Similar to pullulan, the crosslinked pullulan cryogel showed good compatibility with the healthy fibroblast cells. These results support that pullulan cryogels can be used as drug carriers because of their perfect biocompatibility and porous structure. In addition, the cytotoxicity of drug-loaded pullulan-cipro cryogels weighing 1 mg showed slightly toxic behavior toward the fibroblast cells over 24 h of incubation time with 66% ± 9% cell viability. According to the drug delivery study, pullulan-cipro cryogel released 41.3 ± 3.7 μg/mg ciprofloxacin within 24 h. This value indicates that 1 mg of drug-loaded cryogel in contact with bacteria in 100 μL of growth medium could release approximately 0.413 mg/mL cipro. There was no statistical difference between the pullulan cryogel and the control, even at 1 mg. The drug-loaded pullulan-cipro cryogels were not statistically different from the control up to 0.25 mg. It can be said that the drug-loaded pullulan-cipro cryogel was safe against fibroblast cells up to 0.25 mg and slightly toxic dependent on the toxicity of released cipro. The prepared pullulan cryogels, with their possible reassuring biological activities, are promising candidates for use in pharmaceutic and biomedical applications.

## 4. Conclusions

In this study, pullulan cryogels crosslinked with DVS were synthesized for the first time in the absence of a different polymer or composite material. These super-macroporous pullulan cryogels had interconnected pores with variable size ranging from 1 to 200 µm. This porosity expectation, which is crucial for drug loading and release studies, can be controlled by the amount of crosslinker. The hydrolytic degradation capability of the synthesized pullulan cryogels was directly correlated with the pH of the medium. The pullulan cryogels exhibited higher degradation capability in basic medium (in pH 9.0 buffer solution) compared to acidic (in pH 1.0 buffer solution) and neutral medium (in pH 7.4 buffer solution) in the same time period, losing 31% ± 2% of their initial weight in basic medium within 28 days. Pullulan, as a natural polysaccharide, is a promising biomolecule for biomedical applications due to its inherent advantages, e.g., biodegradability, nontoxicity, and biocompatibility. The use of pullulan cryogel, taking advantage of the biodegradable and biocompatible properties of pullulan, as a drug carrier material was tested by loading the cryogel with ciprofloxacin antibiotic as a model drug. The pullulan cryogel was loaded with 105.40 ± 2.6 µg/mg drug and released 41.38% ± 3.58% of the loaded drug within 24 h. The antibacterial activities of pullulan-cipro cryogel were determined against *E. coli* and *S. aureus* strains, revealing that only 0.1 mg of antibiotic-loaded pullulan-cipro cryogel was highly effective in killing Gram-negative *E. coli* and Gram-positive *S. aureus* strains via total eradication within just 2 h of interaction. In addition, pullulan-cipro cryogels had a slightly toxic effect on L929 fibroblast cells, even at 10-fold higher concentrations, for bacterial colony inhibition. Therefore, pullulan cryogels can be utilized as support and transport materials for therapeutics (e.g., ciprofloxacin antibiotic), providing significant advantages with a controllable drug delivery profile, improved therapeutic efficiency, and long-lasting effect with good biocompatibility.

## Figures and Tables

**Table 1:** The determined S%, P%, M%, and V_P_ values for pullulan cryogels.

Material	S %	P%	M%	V_P_ (mL/g)
Pullulan cryogel	588 ± 22	75 ± 0.3	85 ± 0.5	5.0 ± 0.2

## Data Availability

The data that support the findings of this study are available upon reasonable request from the authors.
